# Hepcidin levels in diabetes mellitus and polycystic ovary syndrome

**DOI:** 10.1111/dme.12262

**Published:** 2013-08-19

**Authors:** A H Sam, M Busbridge, A Amin, L Webber, D White, S Franks, N M Martin, M Sleeth, N A Ismail, N Mat Daud, D Papamargaritis, C W Le Roux, R S Chapman, G Frost, S R Bloom, K G Murphy

**Affiliations:** 1 Section of Investigative Medicine, Imperial College London, Hammersmith HospitalLondon, UK; 2 Department of Clinical Biochemistry, Imperial College Healthcare NHS Trust, Charing Cross HospitalLondon, UK; 3 Institute of Reproductive and Developmental Biology, Imperial College London, Hammersmith HospitalLondon, UK

## Abstract

**Aim:**

Increased body iron is associated with insulin resistance. Hepcidin is the key hormone that negatively regulates iron homeostasis. We hypothesized that individuals with insulin resistance have inadequate hepcidin levels for their iron load.

**Methods:**

Serum concentrations of the active form of hepcidin (hepcidin-25) and hepcidin:ferritin ratio were evaluated in participants with Type 2 diabetes (*n* = 33, control subjects matched for age, gender and BMI,*n* = 33) and participants with polycystic ovary syndrome (*n* = 27, control subjects matched for age and BMI,*n* = 16). To investigate whether any changes observed were associated with insulin resistance rather than insulin deficiency or hyperglycaemia per se, the same measurements were made in participants with Type 1 diabetes (*n* = 28, control subjects matched for age, gender and BMI,*n* = 30). Finally, the relationship between homeostasis model assessment of insulin resistance and serum hepcidin:ferritin ratio was explored in overweight or obese participants without diabetes (*n* = 16).

**Results:**

Participants with Type 2 diabetes had significantly lower hepcidin and hepcidin:ferritin ratio than control subjects (*P *< 0.05 and *P *< 0.01, respectively). Participants with polycystic ovary syndrome had a significantly lower hepcidin:ferritin ratio than control subjects (*P *< 0.05). There was no significant difference in hepcidin or hepcidin:ferritin ratio between participants with Type 1 diabetes and control subjects (*P *= 0.88 and *P *= 0.94). Serum hepcidin:ferritin ratio inversely correlated with homeostasis model assessment of insulin resistance (*r* = –0.59, *P < *0.05).

**Conclusion:**

Insulin resistance, but not insulin deficiency or hyperglycaemia per se, is associated with inadequate hepcidin levels. Reduced hepcidin concentrations may cause increased body iron stores in insulin-resistant states.

## What's new?

- This is the first study comparing serum hepcidin levels in people with diabetes (Type 1 and Type 2) against weight-matched control subjects.
- People with Type 2 diabetes had significantly lower serum hepcidin and hepcidin:ferritin ratio than weight-matched control subjects.
- There was a significant negative correlation between serum hepcidin:ferritin ratio and homeostasis model assessment of insulin resistance (a surrogate of insulin resistance).
- We hypothesize that inadequate hepcidin levels for the iron load in people with Type 2 diabetes link increased body iron and insulin resistance.

## Introduction

Evidence suggests that iron plays a role in the pathogenesis of Type 2 diabetes mellitus 1. Iron influences glucose metabolism, even in the absence of significant iron overload 2. Mildly elevated body iron stores are associated with increased fasting serum insulin and blood glucose 3. Lowering iron stores by venesection increases peripheral insulin sensitivity in patients with high-ferritin Type 2 diabetes 4.

Hepcidin is the key hormone regulating iron homeostasis. It is a 25-amino-acid peptide predominantly synthesized in the liver 5,6. Hepatic secretion of hepcidin in response to iron overload negatively regulates iron homeostasis. Hepcidin prevents iron efflux from enterocytes, macrophages and hepatocytes into the plasma by inducing internalization and degradation of the iron exporter ferroportin in these cells 7.

The underlying mechanism for the increased body iron stores in conditions of insulin resistance is unclear. The hepcidin:ferritin ratio has been used as a marker of the adequacy of hepcidin production for a given iron load 8,9. We investigated whether insulin resistance is related to a decrease in hepcidin:ferritin ratio, and thus to inadequate hepcidin levels. Understanding the mechanism underpinning the iron overload associated with insulin resistance may help develop novel therapies for Type 2 diabetes.

## Methods

### Subjects

All studies were approved by the Local Research and Ethics Committee and carried out according to the principles of the Declaration of Helsinki. BMI was calculated from weight and height measurements (kg/m^2^). Blood samples were drawn from participants with Type 2 diabetes, polycystic ovary syndrome and Type 1 diabetes and control subjects at Imperial College Healthcare NHS Trust. All recruited participants with polycystic ovary syndrome satisfied the Rotterdam diagnostic criteria 10. The control subjects for women with polycystic ovary syndrome were women matched for age and BMI. All participants had non-fasting blood samples collected in ethylenediamine tetraacetic acid tubes for measurement of full blood count and HbA_1c_ and in containers without any anticoagulant for serum separation and measurement of hepcidin, ferritin and creatinine. The timing of blood samples collected from the participants with diabetes or polycystic ovary syndrome and control subjects were standardized to exclude the possible confounding effect of the diurnal variation in serum hepcidin levels 11.

As fasting glucose and insulin measurements were not available to calculate homeostasis model assessment of insulin resistance (HOMA-IR) values in these participants, we took further samples from a group of overweight or obese participants without diabetes to investigate the relationship between HOMA-IR and hepcidin:ferritin ratio. Exclusion criteria (intercurrent or chronic medical or psychiatric illness, pregnancy, alcohol or substance abuse) were identified by clinical assessment and examination of medical notes.

### Analytical measurements

Active serum hepcidin-25 levels were measured using a radioimmunoassay developed in-house 11. Haematological and biochemical indices (Table1) were analysed using standard commercial methods on the Abbott Architect ci8000SR (Abbott Diagnostics, Dublin, Ireland) and Sysmex 1000 (Sysmex, Milton Keynes, UK) platforms. HbA_1c_ was measured using a standard commercial method on the Menarini HA-8160 analyser (Menarini Pharma, High Wycombe, UK). HOMA-IR was calculated from mean fasting glucose and insulin levels (insulin × glucose/22.5) measured using standard commercial methods on the Abbott Architect ci8000SR platform and the Abbott Axsym platform (Abbott Diagnostics).

**Table 1:** Clinical and biochemical data in participants with Type 2 diabetes mellitus (study 1), polycystic ovary syndrome (study 2), Type 1 diabetes mellitus (study 3) and control subjects

	Control(*n* = 33)	Study 1 Type 2 diabetes (*n* = 33)	Control (*n* = 16)	Study 2 Polycystic ovary syndrome (*n* = 27)	Control (*n* = 30)	Study 3 Type 1 diabetes (*n* = 28)
Age	48.00 (40.00–62.50)	53.08 (47.23–59.97)	37.81 (32.76–40.01)	30.93 (27.64–37.93)	40.13 (34.73–47.97)	44.67 (31.34–57.34)
Female:male	10:23	10:23	—	—	17:13	17:11
BMI (kg/m^2^)	31.03 ± 1.82	32.23 ± 1.09	30.16 ± 1.93	30.33 ± 1.50	24.59 ± 0.42	23.80 ± 0.53
Creatinine (μmol/l)	75.09 ± 2.36	77.04 ± 2.37	69.49 ± 3.12	65.44 ± 1.35	70.98 ± 2.58	69.21 ± 1.83
HbA_1c_ (mmol/mol)%	**37.51 ± 0.93** **(5.58 ± 0.09)**	**73.17 ± 4.12**† **(8.85 ± 0.38)**	34.63 ± 1.02 (5.32 ± 0.09)	43.98 ± 3.93 (6.17 ± 0.36)	**35.34 ± 0.91** **(5.38 ± 0.08)**	**65.46 ± 2.75**† **(8.14 ± 0.25)**
Haemoglobin (g/dl)	14.24 ± 0.31	14.10 ± 0.22	12.93 ± 0.28	13.50 ± 0.17	13.95 ± 0.28	14.10 ± 0.25
Ferritin (μg/l)	83.00 (42.50–127.60)	97.00 (45.50–183.50)	41.00 (20.00–72.75)	55.00 (40.00–86.00)	64.50 (35.25–105.50)	64.50 (33.50–125.00)
Transferrin saturation (%)	23.41 (17.87–30.75)	20.81 (15.50–25.26)	30.53 (19.52–35.00)	27.18 (18.13–36.54)	26.28 (24.09–34.00)	27.41 (19.87–39.20)
Hepcidin (ng/ml)	**33.00 (18.05–54.00)**	20.00 (10.00–41.00)*	31.50 (8.23–54.00)	16.70 (8.60–27.00)	17.00 (9.83–46.25)	24.50 (9.48–52.25)
Hepcidin:ferritin ratio	**0.45 (0.26–0.58)**	**0.22 (0.15–0.32)**†	**0.55 (0.33–1.04)**	0.29 (0.20–0.47)*	0.32 (0.18–0.58)	0.33 (0.19–0.75)
hsCRP (mg/l)	1.80 (0.45–7.20)	3.50 (1.20–6.15)	1.65 (0.75–4.40)	3.40 (1.10–5.40)	0.90 (0.43–4.75)	1.90 (0.73–4.03)

Data are represented as mean ± sem or median (interquartile range).

Significantly different values are shown in bold; *P < 0.05, †*P *< 0.01.

### Statistical analysis

Data are represented as the mean ± standard error of mean (sem) or median (interquartile range). The differences between groups were assessed using the Student *t*-test for parametric data or the Mann–Whitney *U*-test for non-parametric data. The association between hepcidin and HOMA-IR was assessed by Spearman's correlation coefficient. Prism version 5.1 software (GraphPad Software, San Diego, CA, USA) was used for all statistical analyses. A *P*-value < 0.05 was considered statistically significant.

## Results

The clinical and biochemical characteristics of participants with Type 2 diabetes (study 1), polycystic ovary syndrome (study 2), Type 1 diabetes (study 3) and weight-matched control subjects are summarized in Table1. Participants with Type 2 diabetes (*n* = 33) had significantly lower median hepcidin-25 levels (*P *< 0.05) and median hepcidin:ferritin ratio (*P *< 0.01) than weight-matched control subjects (*n* = 33). There was a trend towards reduced median hepcidin-25 levels in participants with polycystic ovary syndrome (*n* = 27) compared with weight-matched control subjects (*n* = 16); however, this did not reach statistical significance. Participants with polycystic ovary syndrome had a significantly lower median hepcidin:ferritin ratio than control subjects (*P *< 0.05). Seven participants with polycystic ovary syndrome (26%) had regular cycles. Exclusion of these individuals did not change the median hepcidin:ferritin ratio. The median hepcidin:ferritin ratio for all participants with polycystic ovary syndrome and for those with polycystic ovary syndrome and oligomenorrhoea was 0.29 (0.20–0.47) and 0.29 (0.20–0.53), respectively.

There was no significant difference in median serum hepcidin-25 levels (*P *= 0.88) and median hepcidin:ferritin ratio (*P *= 0.94) between participants with Type 1 diabetes (*n* = 28) and weight-matched control subjects (*n* = 30). The median duration of Type 1 diabetes was 16 (5–29) years.

HOMA-IR, a marker of insulin resistance, and serum hepcidin:ferritin ratio were measured in 16 overweight or obese participants without diabetes (four men and 12 women). The median age and mean BMI of the subjects were 34 (27.5–40.75) years and 29.6 ± 0.9 kg/m^2^, respectively. There was a significant negative correlation between hepcidin:ferritin ratio and HOMA-IR (*r* = −0.59, *P < *0.05).

## Discussion

We report for the first time that participants with Type 2 diabetes have a significantly lower hepcidin and hepcidin:ferritin ratio compared with weight-matched control subjects. Reduced hepcidin:ferritin ratio indicates inadequate hepcidin levels in response to the degree of iron load. Reduced hepcidin concentrations may contribute to iron overload by increasing the intestinal absorption of iron. Moderately increased iron levels, much lower than those found in haemochromatosis, are associated with elevated risk of Type 2 diabetes 1. The serum hepcidin:ferritin ratio was also significantly lower in participants with polycystic ovary syndrome than in weight-matched control subjects. This is consistent with a previous study reporting increased ferritin:hepcidin molar ratios in patients with polycystic ovary syndrome 12.

It has been shown that insulin resistance can lead to a down-regulation of hepcidin expression in rats on a high-fat/high-energy diet 13. Whether the reduction in hepcidin concentrations in participants with Type 2 diabetes is a primary event or secondary to insulin resistance remains to be determined. However, the relationship between iron metabolism and insulin resistance is bidirectional, as iron accumulation favours insulin resistance and insulin resistance may in turn result in increased body iron stores 2. Therefore, a reduction in hepcidin levels leading to increased intestinal absorption of iron can exacerbate insulin resistance, even if it is initially secondary to insulin resistance.

Serum hepcidin and the hepcidin:ferritin ratio was not significantly different in participants with Type 1 diabetes compared with control subjects. This suggests that the inadequate hepcidin levels observed in participants with Type 2 diabetes are unlikely to be a consequence of insulin deficiency alone or hyperglycaemia per se. Thus, it is likely that the reduced hepcidin concentrations in participants with Type 2 diabetes are associated with insulin resistance and/or hyperinsulinaemia. Consistent with this, we also showed a significant negative correlation between HOMA-IR, a surrogate of insulin resistance, and hepcidin:ferritin ratio in overweight or obese participants without diabetes.

Impaired urinary excretion may lead to increased circulating levels of hepcidin. It has been shown that plasma hepcidin levels are elevated in renal disease and that these increases in hepcidin concentration reflect the degree of renal impairment 14. Obesity is also associated with elevated levels of serum hepcidin 15. The participants in the only previous study of hepcidin levels in patients with Type 2 diabetes had significantly higher serum creatinine and higher BMI than control subjects 16, suggesting that the higher hepcidin levels in patients with Type 2 diabetes were secondary to differences in renal function or BMI. However, in the current study, control subjects were matched for serum creatinine and BMI, excluding the confounding effects of renal impairment and body weight on serum hepcidin. In addition, we measured the serum hepcidin:ferritin ratio in the two groups to allow an assessment of the appropriateness of hepcidin concentrations in response to the participants' iron burden.

The gene encoding hepcidin has also been reported to be up-regulated by inflammation 17. In our study, high-sensitivity C-reactive protein (hsCRP) levels did not differ significantly between participants with or without Type 2 diabetes, making acute inflammation as a confounding factor unlikely (Table1). In addition, the fact that participants with Type 2 diabetes and polycystic ovary syndrome had lower circulating hepcidin levels than control subjects suggests that inflammation is not the predominant factor regulating hepcidin levels in these individuals. Indeed, a recent population study has shown that CRP is not a significant determinant of hepcidin-25 18.

It is tempting to speculate that the reduction in hepcidin levels observed in individuals with Type 2 diabetes is attributable to reduced production associated with hyperinsulinaemia. Insulin inhibits production of liver proteins, including sex hormone binding globulin and insulin-like growth factor-binding protein 1 19. However, the differences observed in our study may reflect changes in hepcidin clearance, or in both production and clearance.

In summary, participants with conditions associated with insulin resistance (i.e. Type 2 diabetes and polycystic ovary syndrome) had inadequate hepcidin concentrations for their iron load compared with weight-matched control subjects. The results of this small, preliminary study require verifying in larger studies, and the significance of the altered hepcidin levels in the obese are currently unclear, complicating interpretation. However, given increased iron may play a role in the pathogenesis of Type 2 diabetes, these findings have implications for the treatment of insulin resistance and Type 2 diabetes.

## Funding sources

The Section is supported by an Integrative Mammalian Biology (IMB) Capacity Building Award, an FP7-HEALTH-2009-241592 EurOCHIP grant, and funding from the NIHR Biomedical Research Centre Funding Scheme, the BBSRC and the MRC. AH Sam was funded by a Wellcome Trust Research Training Fellowship.

## Competing interests

None declared.
